# Complete de novo assembly of *Wolbachia* endosymbiont of *Drosophila willistoni* using long-read genome sequencing

**DOI:** 10.1038/s41598-024-68716-w

**Published:** 2024-08-01

**Authors:** Jodie Jacobs, Anne Nakamoto, Mira Mastoras, Hailey Loucks, Cade Mirchandani, Lily Karim, Gabriel Penunuri, Ciara Wanket, Shelbi L. Russell

**Affiliations:** 1 Department of Biomolecular Engineering, University of California, Santa Cruz, CA USA; 2 Genomics Institute, University of California, Santa Cruz, CA USA; 3 Department of Ecology and Evolutionary Biology, University of California, Santa Cruz, CA USA

**Keywords:** *Wolbachia*, *Drosophila*, Symbiosis, Genomics, Genomics, Symbiosis

## Abstract

*Wolbachia* is an obligate intracellular α-proteobacterium, which commonly infects arthropods and filarial nematodes. Different strains of *Wolbachia* are capable of a wide range of regulatory manipulations in their diverse hosts, including the modulation of host cellular differentiation to influence host reproduction. The genetic basis for the majority of these phenotypes is unknown. The *w*Wil strain from the neotropical fruit fly, *Drosophila willistoni*, exhibits a remarkably high affinity for host germline-derived cells relative to the somatic cells. This trait could be leveraged for understanding how *Wolbachia* influences the host germline and for controlling host populations in the field. To further the use of this strain in biological and biomedical research, we sequenced the genome of the *w*Wil strain isolated from host cell culture cells. Here, we present the first high quality Nanopore assembly of *w*Wil, the *Wolbachia* endosymbiont of *D. willistoni*. Our assembly resulted in a circular genome of 1.27 Mb with a BUSCO completeness score of 99.7%. Consistent with other insect-associated *Wolbachia* strains, comparative genomic analysis revealed that *w*Wil has a highly mosaic genome relative to the closely related *w*Mel and *w*Au strains from *Drosophila melanogaster* and *Drosophila simulans*, respectively.

## Introduction

*Wolbachia* is a gram-negative α-proteobacterium and is found as an endosymbiont in many arthropods and nematodes with a diverse range of effects on host phenotypes^1,2^. *Wolbachia* are maternally transmitted through host oocytes to the developing embryo^1^. Some *Wolbachia* strains manipulate host reproduction to promote their transmission to the next generation of hosts^2^. *Wolbachia* strains have strong affinities for host germline tissues, enabling efficient colonization of the oocytes for transmission to the next generation of hosts^3^. The *Wolbachia* strain from the neotropical fruit fly *Drosophila willistoni, w*Wil, selectively infects the host germline^4,5^. This unique tropism could be informative for understanding how *Wolbachia* localizes to and regulates the host germline, with implications for vectorizing *Wolbachia* infections for biological control mechanisms.

The strong affinity of *w*Wil for host germline cells is unique in comparison to closely related *Wolbachia* strains. Phylogenetic comparisons based on amplification of the *wsp* and *ftsZ* genes by PCR indicate that *w*Wil is closely related to the *w*Au strain found in *Drosophila simulans*^4^. However, unlike *w*Au, which infects both germline and somatic tissues in *D. simulans*, *w*Wil is enriched in the primordial germline cells of *D. willistoni* embryos^4^. As with many *Wolbachia strains, w*Wil exhibits strict maternal transmission in laboratory lines.

Given that all *Wolbachia* strains must navigate to the female host germline for transmission to the next host generation, the *w*Wil strain genome offers insight into the genomic repertoire enabling this phenotype. Despite the availability of numerous *Wolbachia* genomes in public databases, a complete *w*Wil genome is lacking. Here we present the first high-quality de novo assembly of *w*Wil, which we obtained from Nanopore sequencing of *w*Wil-infected *D. melanogaster* cell culture cells. We perform comparative genomics analyses of the *w*Wil, *w*Mel, and *w*Au genomes to identify differences that can provide insights into the mechanisms underlying *w*Wil’s germline-specific distribution. This genomic resource will provide insight into the essential genes required for efficient germline transmission in *Wolbachia*-based biological control strategies.

## Methods

### *w*Wil genome assembly

We obtained *w*Wil-infected *Drosophila willistoni* flies collected from Guadeloupe Island from the Drosophila Species Stock Center at University of California, San Diego (14,030–0811.24). These are now available from Cornell University (Powell Gd-H4-1). We isolated *w*Wil from *w*Wil-infected *D. willistoni* embryos^6^ and introduced *w*Wil to immortalized *Drosophila melanogaster* JW18 cell culture cells with the shell vial technique^7^. Briefly, we isolated *w*Wil from infected embryos by filtration through a sterile 5 µm syringe filter, followed by a secondary filtration through a 1.2 µm filter. We pipetted the *w*Wil-containing lysate into a shell vial seeded with a monolayer of JW18 cells 24 h earlier, and gently centrifuged the mixture to force the *w*Wil into the JW18 cells at 2500 × *g* for 1 h at 15 °C. We allowed the infection to stabilize by maintaining the culture for three months at 23 °C. Confluent cultures were sampled for genomic DNA extraction and library preparation. Our *w*Wil-infected *D. melanogaster* cell culture system offered multiple advantages for generating a complete de novo genome assembly over directly sequencing *w*Wil-infected *D. willistoni* flies. *w*Wil replicates to higher titer in vitro than in its insect hosts, as evidenced by the high proportions of *Wolbachia*-derived reads in our datasets relative to in vivo data obtained previously (GCA_000153585.1). Additionally, it is significantly easier to extract the high quality long genomic DNA necessary for long read sequencing from cell culture cells than it is from whole fly tissues.

To prepare *w*Wil genomic DNA for Nanopore library preparation and sequencing, 1.2 mL (at ~ 2e6 cells/mL) of cells were pelleted by centrifugation at 16,000xg for 10 min at 4 °C. Following supernatant removal, DNA was extracted using the Wizard HMW DNA Extraction kit (Promega #A2920, Lot: 0,000,575,812). Libraries were prepared with the Native Barcoding Kit V14 for Nanopore MinION R10 (Oxford Nanopore Technologies Cat #SQK-NBD114-24, Lot: NDP1424.10.0010) and sequenced on the Nanopore MinION Mk1B with a MinION R10 Version flow cell (FLO-MIN-114, Lot:11,003,064). We used Oxford Nanopore’s MinKNOW v23.07.8 software to live basecall with Guppy v7.0.8 (Fast model, read splitting ON). We set the minimum read length to 200 bp and stopped sequencing after 36 h. This resulted in 3.65 M reads with an estimated N50 of 1.11 kb and 2.6 Gb called with a minQ of 8.

Prior to genome assembly, we preprocessed the raw nanopore reads to remove host-derived sequences. Reads were aligned to the *D. melanogaster* reference genome (dmell-all-r6.46)^8^ with bwa-mem^9^ v0.7.17. We used samtools^10^ v1.6 to sort and index the alignment and remove reads which aligned to the host genome (samtools view -b -f 4). The remaining reads were output with bedtools^11^ v2.31.1 bamtofastq. Artifacts in MinKNOW resulted in duplicated Nanopore reads. We removed read duplicates with SeqKit^12^ rmdup v2.7.0 and performed a de novo assembly of the *w*Wil genome with Flye^13^ v2.9 (preset, –nano-hq). We screened the assembly for foreign genomic and adapter contamination using the NCBI Foreign Contamination Screen (FCS) toolkit version 0.5.0. We ran FCS-GX^14^ (taxa ID 953) and FCS-adaptor (run with–prok flag) which both found no evidence of contamination.

### Genome polishing, annotation, and quality assessment

We generated Illumina short read whole genome sequence data from JW18 cell culture cells stably infected with *w*Wil to polish the Nanopore assembly. Genomic DNA was obtained as described above for Nanopore sequencing. Illumina libraries were made following the Tn5 protocol^15^ and sequenced on a NovaseqX by the Duke Sequencing and Genomic Technologies Core Facility. Illumina reads were aligned to the *w*Wil assembly and *D. melanogaster* reference^8^ (dmel6) simultaneously using bwa-mem^9^ with default settings. Optical and PCR duplicates were marked with sambamba^16^. The reads aligning to dmel6 were discarded. The remaining reads were converted back to fastq format using samtools^10^ fastq, and then re-aligned to the *w*Wil genome using minimap2^17^ v2.26 with the settings-ax sr –cs –eqx. Reads with a gap-compressed mismatch ratio exceeding 0.04 were filtered out to remove mismapping and excess noise prior to polishing. The tool Pilon^18^ v1.24 was run on these filtered alignments using default settings, producing the final polished assembly.

**Figure 1:**
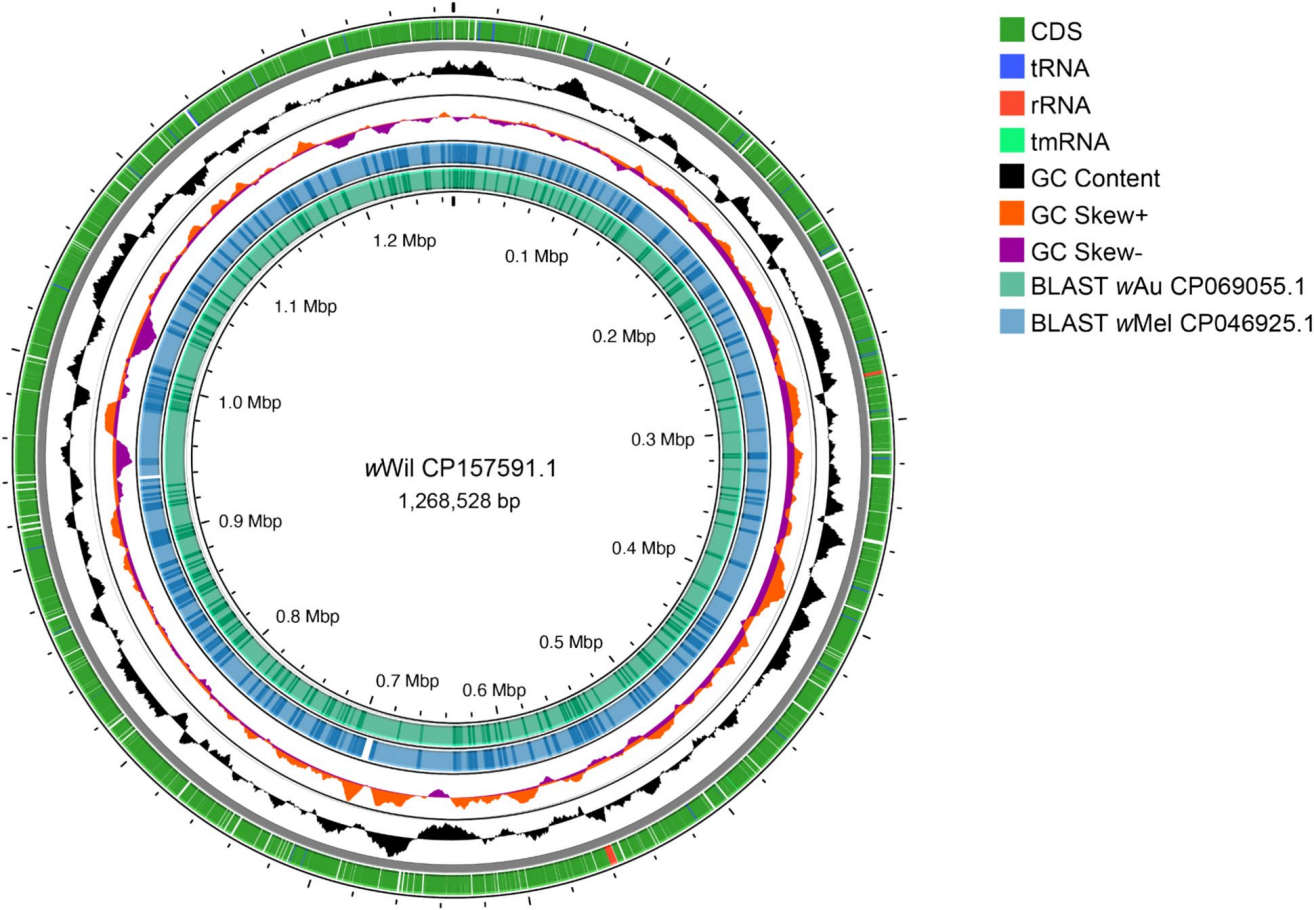
Map of the *Wolbachia w*Wil genome prepared using Proksee^22^. Circles in order from outer to inner show the following features: the position of coding sequences (CDS), open reading frames (ORF), tmRNA, tRNA, and rRNA genes (circle 1). GC content (circle 2) and GC skew plotted as the deviation from the average for the entire sequence (circle 3). The positions of BLAST hits detected through BLASTn comparisons of *w*Mel CP046925.1^32^ and *w*Au CP069055.1^33^ are shown in transparent blue and green. Sites in the *w*Wil genome that map to multiple positions in the *w*Mel and *w*Au genomes are indicated by the darker, overlapping colors (circles 4 and 5).

We assessed the quality of the polished assembly with BUSCO^19^ and annotated the genome with a standard workflow. BUSCO scores were calculated using the rickettsiales_odb10 database and v5.7.0. Default parameters were used for all software unless otherwise specified. We annotated the *w*Wil genome with Prokka^20^ v1.1.1 (kingdom:bacteria) and the NCBI Prokaryotic Genome Annotation Pipeline (PGAP) v6.7^21^ to identify coding sequences (CDS), tRNAs, rRNAs, and tmRNA. GC Content and GC Skew were calculated with Proksee^22^ v1.1.2. We then aligned the *w*Wil genome against the *w*Mel (CP046925.1) and *w*Au (CP069055.1) reference genomes with BLASTn, setting the expected value cut-off at 0.0001. We plotted these annotations with Proksee^22^ v1.1.2 to visualize the annotated genome (Fig. 1).

### Comparative genomic analyses

To place our *w*Wil genome within the *Wolbachia* phylogeny, we gathered a set of 27 circular, chromosome-level genome assemblies from diverse *Wolbachia* supergroups across a broad host range^23^. *Ehrlichia chaffeensis* was used as an outgroup. Genes were annotated using the NCBI Prokaryotic Genome Annotation Pipeline^21^, and groups of orthologous genes (orthogroups) were identified with OrthoFinder2^24^. This produced protein sequence alignments of each orthogroup, and those that were single copy orthologous genes (SCOs) were used to generate a maximum likelihood (ML) phylogeny with IQ-TREE^25^ (1000 bootstrap replicates), rooted on *E. chaffeensis*. Additionally, we utilized BUSCO^19^ analysis to characterize gene presence-absence variation across orthogroups.

Whole genome alignments were performed with progressiveMauve^26^ (snapshot 2015-02-25.1) to identify local collinear blocks of orthologous sequence and identify structural rearrangements. We visualized the breaks in synteny between *w*Wil and both reference genomes by generating dotplots with D-GENIES^27^.

We also performed a brief assessment of putative secreted and membrane-bound proteins that could play a role in the *Wolbachia*-host interaction. Proteins containing a signal peptide sequence were identified by SignalP^28^. Transmembrane protein domains were identified by TMHMM^29^. The subset of proteins with a signal peptide and a transmembrane domain were classified as membrane-bound proteins, while those with a signal peptide but without a transmembrane domain were classified as secreted proteins. We then characterized presence-absence variation of putative secreted and membrane proteins within groups of orthologous genes across strains. Finally, we identified variable sites in all proteins by calculating the Shannon entropy metric^30,31^, and compared the number of high-entropy sites in membrane and secreted proteins versus all proteins in general.

## Results and discussion

### Genome assessments

**Figure 2:**
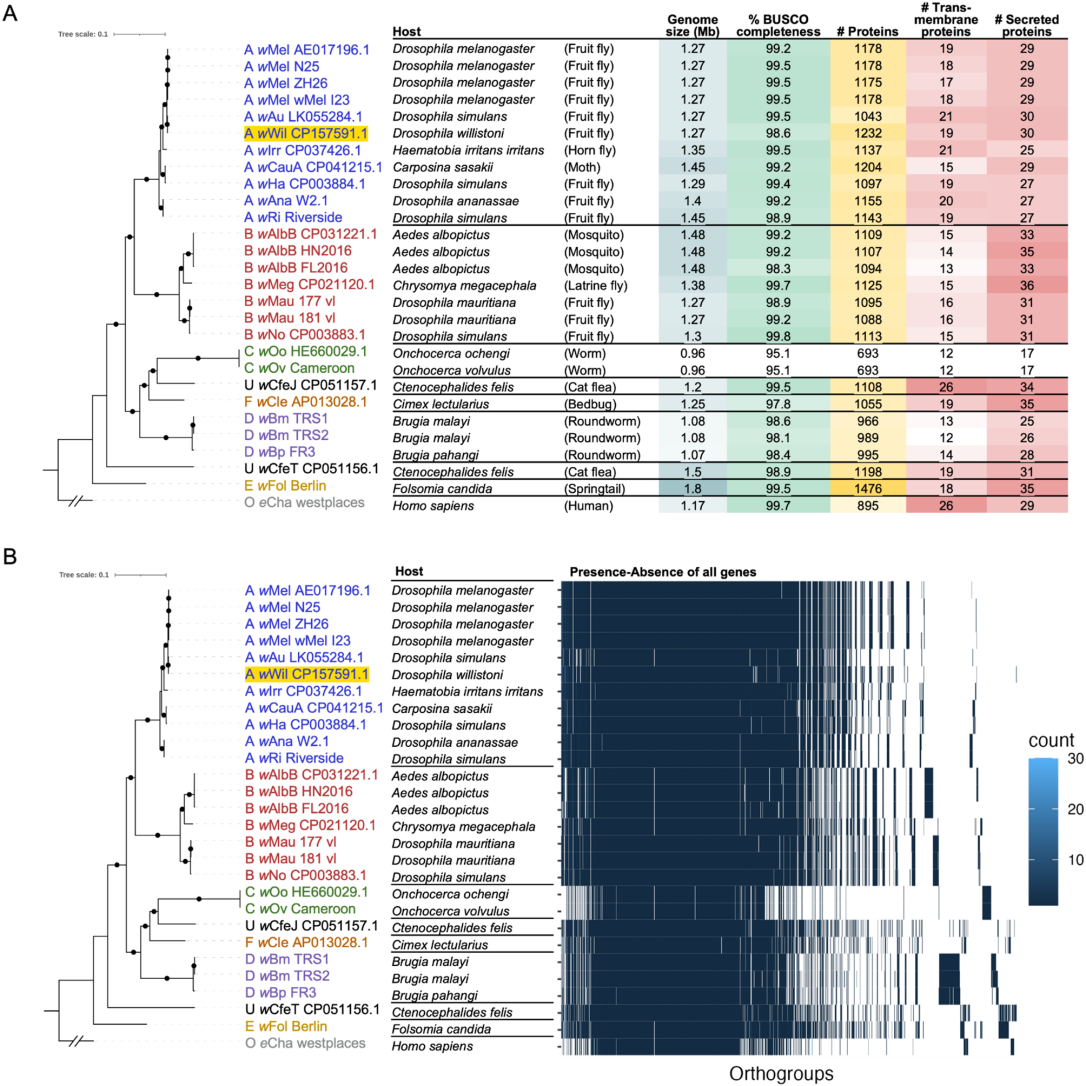
Comparative phylogenomics of *w*Wil among other *Wolbachia* strains. **(a)** ML phylogeny of *Wolbachia* genomes (bootstrap values of 90 or greater are indicated by black circles) based on 470 single-copy orthologous genes (SCOs), with *w*Wil in supergroup A, along with genome metadata: host species and common name, genome size (Mb), BUSCO completeness score (%), total number of proteins, number of putative transmembrane proteins, and number of putative secreted proteins. **(b)** The same phylogeny as in A, with the presence-absence variation of all orthogroups shown. Whitespace indicates the absence of a gene in a particular *Wolbachia* genome.

We generated long-read nanopore data from *w*Wil-infected *D. melanogaster* JW18 cells and assembled a 1.268 Mb genome assembly containing a single circular contig. Our *w*Wil assembly had a high BUSCO completeness score of 98.6% before polishing, which was comparable to the other circular, chromosome-level *Wolbachia* genomes (Fig. 2A and Supplemental Table S1). Polishing produced an improvement in BUSCO score to 99.7%. We annotated the polished *w*Wil genome to identify coding sequences (CDSs), tRNAs, rRNAs, and tmRNA. The polished assembly had a GC content of 35.23% and contained 1302 total genes with 1199 protein coding CDSs, three complete ribosomal RNA genes, 32 tRNAs, and 4 ncRNAs (Table 1).

**Table 1:** *w*Wil CP157591.1 annotation summary statistics prepared by the NCBI Prokaryotic Genome Annotation Pipeline (PGAP) v6.7.

*w*Wil CP157591.1 Annotation summary	
Annotation pipeline	NCBI prokaryotic genome annotation pipeline (PGAP) v6.7
Annotation method	Best-placed reference protein set; GeneMarkS-2 +
Length (bp)	1,268,528
GC Content	35.23%
Genes (total)	1302
CDSs (total)	1261
CDSs (with protein)	1199
Genes (RNA)	41
rRNAs	1, 1, 1 (5S, 16S, 23S)
tRNAs	32
ncRNAs	4
Pseudo Genes (total)	62

### Genome comparisons

**Figure 3:**
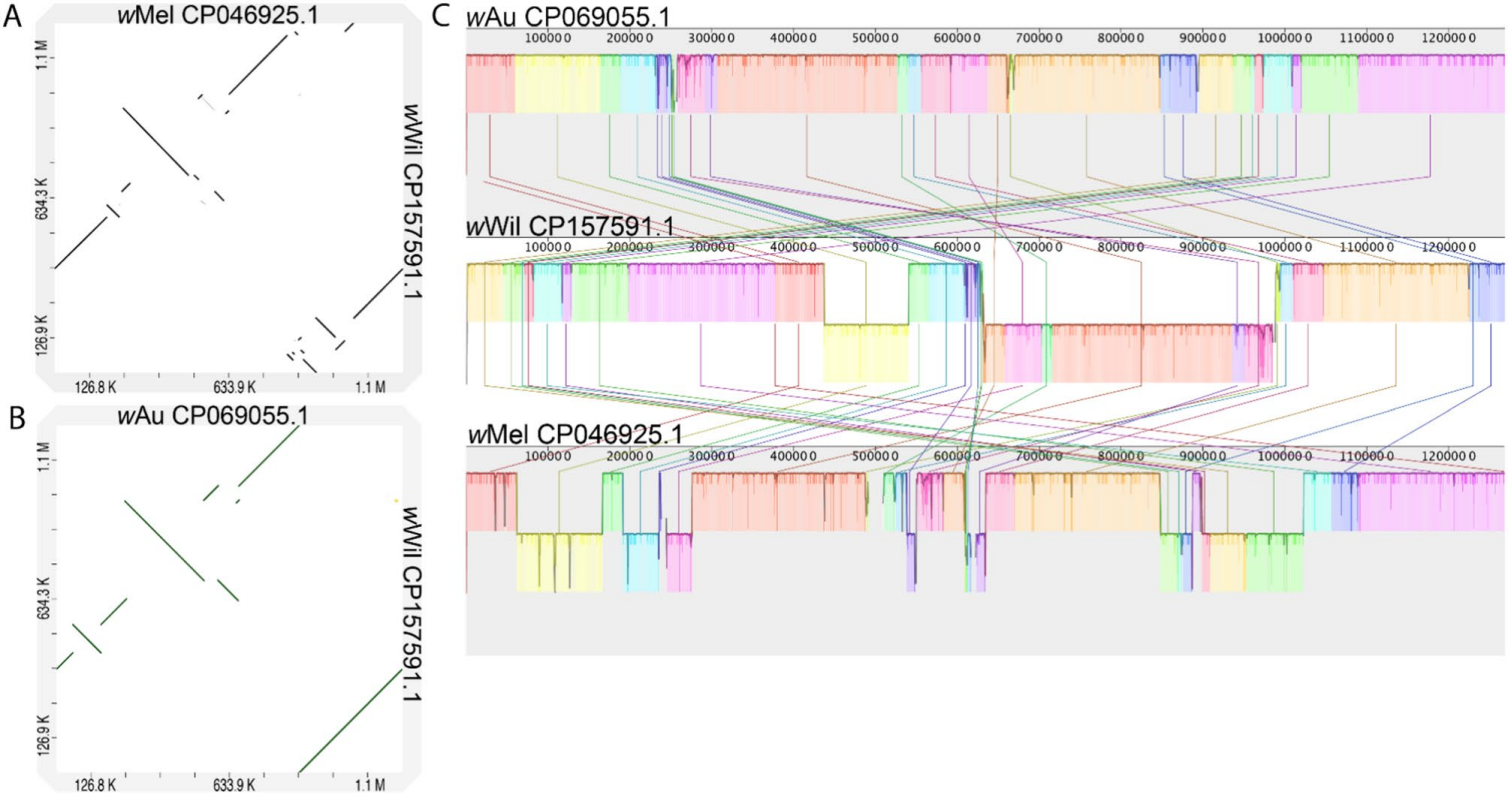
Alignment of reference strains *w*Mel CP046925.1^32^ and *w*Au CP069055.1^33^ with *w*Wil CP15759.1. (**a, b**) Dotplot generated with D-GENIES^27^ (**c**) Mauve alignment showing locally collinear blocks (LCBs) identified along the circular genomes and joined with vertical lines.

To explore the differences between *w*Wil and other *Wolbachia* strains, we performed genome alignments and analyses of orthologous gene content across diverse *Wolbachia*. Our phylogenetic analysis of SCOs from 27 *Wolbachia* genomes revealed that *w*Wil resides in *Wolbachia* supergroup A, alongside *w*Mel, *w*Au, and many other fly-infecting strains (Fig. 2A). Despite being closely related, dotplots revealed genomic rearrangements compared to both *w*Mel (Fig. 3A) and *w*Au (Fig. 3B) with larger regions of homology to *w*Au. Alignment of the *w*Wil genome to the *w*Mel CP046925.1^32^ and *w*Au CP069055.1^33^ reference genomes revealed many breaks in synteny between the genomes (Fig. 3C).

**Figure 4:**
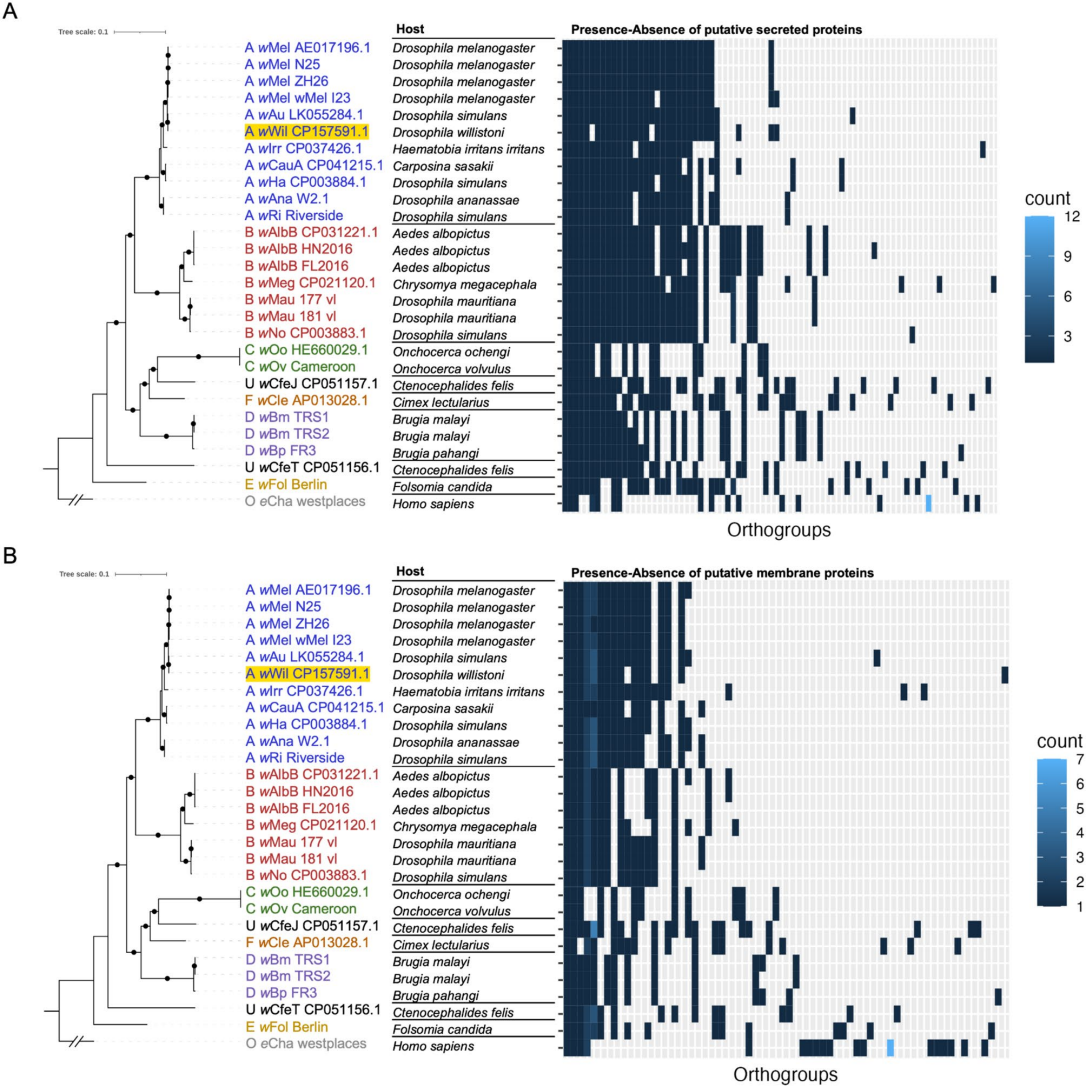
Presence-absence variation of putative **(a)** membrane protein and **(b)** secreted protein genes across orthogroups in *Wolbachia* strains. As in Fig. 2, the absence of a tile indicates the absence of a gene in a particular *Wolbachia* strains.

We then investigated genes that are present in some *Wolbachia* strains but absent in others, and could thus play a role in *Wolbachia’s* adaptation to different hosts. In general, our analysis showed a supergroup-specific pattern of gene presence-absence variation (Fig. 2B), with some sets of genes being unique to particular supergroups, or to individual *Wolbachia* strains. We then looked more specifically at membrane-bound and secreted proteins, which are often implicated in interactions between *Wolbachia* and its host. Just as for all genes, there was a supergroup-specific pattern in presence-absence variation for both membrane-bound and secreted proteins across *Wolbachia* strains (Fig. 4 and Supplemental Tables S2 and S3).

**Figure 5:**
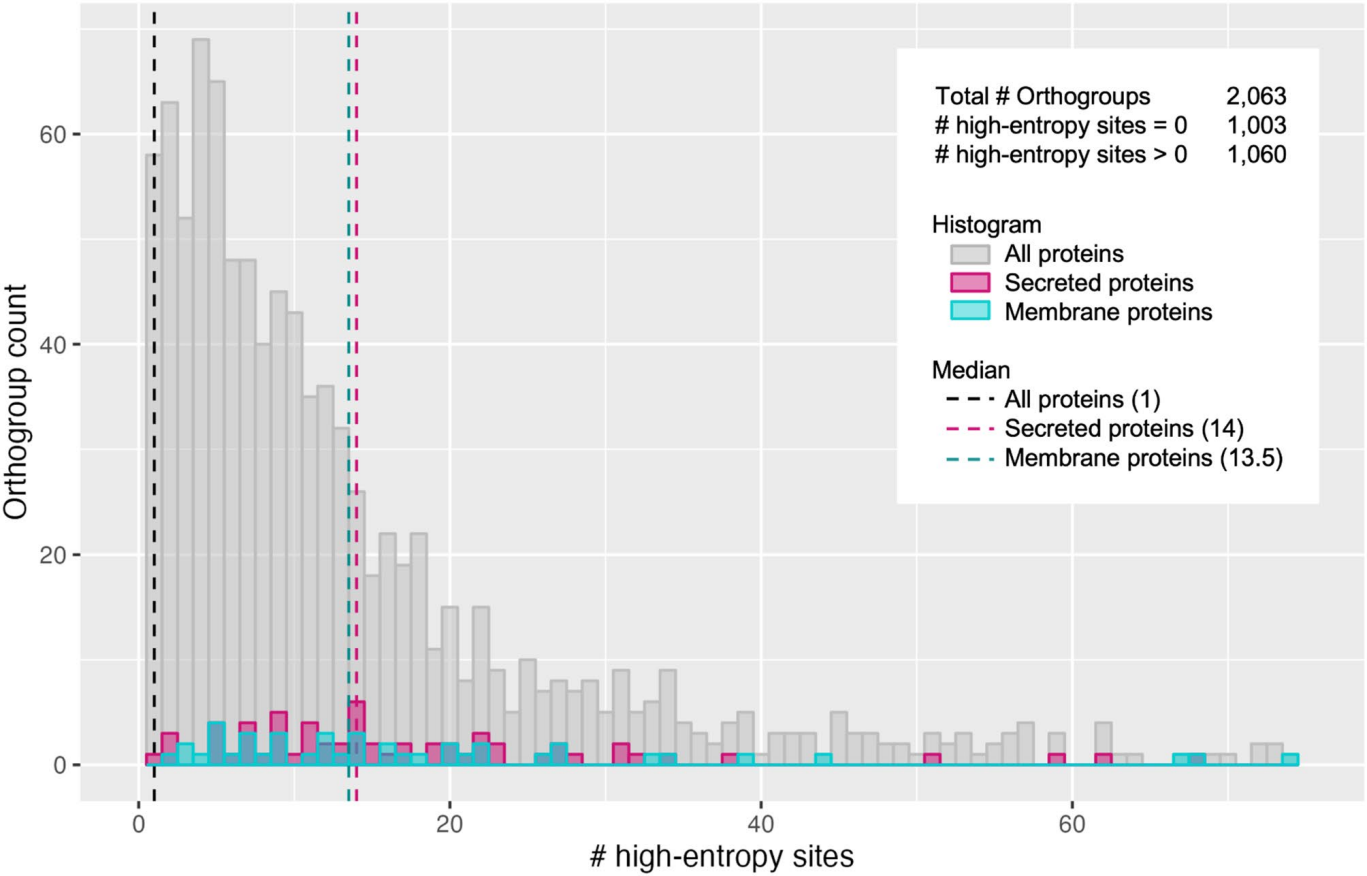
Variability of membrane proteins and secreted proteins compared to all proteins. Shown is a histogram of the distribution of orthogroups across the number of high-entropy (variable) sites in their protein sequence alignment. Orthogroup counts are plotted separately for all proteins (gray), secreted proteins (pink), and membrane proteins (blue), with median number of variable sites represented by dashed lines of the respective colors. There were 1,003 orthogroups that did not contain any variable sites, which are not included in the plot.

Additionally, our analysis of sequence entropy in membrane and secreted protein groups showed that they had many variable sites compared to all proteins in general. The median number of variable sites in an orthogroup across all *Wolbachia* genes was one, while the medians for secreted and membrane proteins were 14 and 13.5 variable sites, respectively (Fig. 5). It is especially of interest to identify secreted proteins, known as effectors, that contain variable sites and may interact with host machinery, resulting in a phenotype of interest. One such phonetype is the rescue of sterile *Sex-lethal* (*Sxl*) mutants, caused by the effector, TomO’s interaction with host mRNA^34^. We find that there are 144 variable sites (~ 10%) in the alignment of TomO orthologs, suggesting rapid evolution of this gene driven by the arms-race between host and symbiont^35–37^. Thus, our results support the idea that TomO plays an important role in the interaction with the host across different *Wolbachia* strains. Other examples include the WalE1 actin-associated protein^38^ and cytoplasmic incompatibility factors (Cifs)^23^, which we also find to have many variable sites (216 or ~ 26% of sites, and 209 or ~ 22% of sites, respectively). Along with these known effectors, there are certainly many more that are yet uncharacterized. Overall, this analysis revealed proteins with many sites that vary across diverse *Wolbachia* strains with a wide host range, and thus provides candidates for further interrogating *Wolbachia*-host interactions at the molecular level.

## Conclusion

Our assembly of the first high-quality *w*Wil strain genome will enable deeper understanding of *Wolbachia* Supergroup A evolution in *Drosophila* hosts and the evolution of germline tropisms. The use of a novel *D. melanogaster* cell line infected with the *w*Wil strain enabled us to obtain high molecular weight *w*Wil gDNA for Nanopore sequencing. The genome assembly and annotation produced from these data will be a vital resource for future investigations on this strain and its germline-specific tropism. Furthermore, the membrane and secreted proteins identified in our analyses inform on candidate genes that may be involved in mediating bacterial-host interactions to promote infection and intracellular persistence.

### Supplementary Information

Supplementary Tables.

## Data Availability

The assembled genome and the raw long and short reads are available in BioProject PRJNA1107195.

## References

[1] Russell, S. L. & Castillo, J. R. Trends in symbiont-induced host cellular differentiation. *Results Probl. Cell Differ.* **69**, 137–176 (2020). 33263871 10.1007/978-3-030-51849-3_5 PMC8025664

[2] Werren, J. H., Baldo, L. & Clark, M. E. Wolbachia: Master manipulators of invertebrate biology. *Nat. Rev. Microbiol.* **6**, 741–751 (2008). 18794912 10.1038/nrmicro1969

[3] Toomey, M. E., Panaram, K., Fast, E. M., Beatty, C. & Frydman, H. M. Evolutionarily conserved *Wolbachia*-encoded factors control pattern of stem-cell niche tropism in Drosophila ovaries and favor infection. *Proc. Natl. Acad. Sci.* **110**, 10788–10793 (2013). 23744038 10.1073/pnas.1301524110 PMC3696799

[4] Miller, W. J. & Riegler, M. Evolutionary dynamics of wAu-like *wolbachia* variants in neotropical *Drosophila* spp.. *Appl. Environ. Microbiol.* **72**, 826–835 (2006). 16391124 10.1128/AEM.72.1.826-835.2006 PMC1352291

[5] Strunov, A., Schmidt, K., Kapun, M. & Miller, W. J. Restriction of *Wolbachia* bacteria in early embryogenesis of neotropical drosophila species via endoplasmic reticulum-mediated autophagy. *mBio* **13**, e03863-e3921 (2022). 35357208 10.1128/mbio.03863-21 PMC9040723

[6] Müller, M. J. *et al.* Reevaluating the infection status by the *Wolbachia* endosymbiont in *Drosophila* Neotropical species from the *willistoni* subgroup. *Infect. Genet. Evol.* **19**, 232–239 (2013). 23906981 10.1016/j.meegid.2013.07.022

[7] Dobson, S. L., Marsland, E. J., Veneti, Z., Bourtzis, K. & O’Neill, S. L. Characterization of *Wolbachia* host cell range via the in vitro establishment of infections. *Appl. Environ. Microbiol.* **68**, 656–660 (2002). 11823204 10.1128/AEM.68.2.656-660.2002 PMC126719

[8] Hoskins, R. A. *et al.* The release 6 reference sequence of the *Drosophila* melanogaster genome. *Genome Res.* **25**, 445–458 (2015). 25589440 10.1101/gr.185579.114 PMC4352887

[9] Li, H. *Aligning Sequence Reads, Clone Sequences and Assembly Contigs with BWA-MEM*. Preprint at 10.48550/arXiv.1303.3997 (2013).

[10] Li, H. *et al.* The sequence alignment/map format and SAMtools. *Bioinformatics* **25**, 2078–2079 (2009). 19505943 10.1093/bioinformatics/btp352 PMC2723002

[11] Quinlan, A. R. & Hall, I. M. BEDTools: A flexible suite of utilities for comparing genomic features. *Bioinformatics* **26**, 841–842 (2010). 20110278 10.1093/bioinformatics/btq033 PMC2832824

[12] Shen, W., Le, S., Li, Y. & Hu, F. SeqKit: A cross-platform and ultrafast toolkit for FASTA/Q file manipulation. *PLOS ONE* **11**, e0163962 (2016). 27706213 10.1371/journal.pone.0163962 PMC5051824

[13] Kolmogorov, M., Yuan, J., Lin, Y. & Pevzner, P. A. Assembly of long, error-prone reads using repeat graphs. *Nat. Biotechnol.* **37**, 540–546 (2019). 30936562 10.1038/s41587-019-0072-8

[14] Astashyn, A. *et al.* Rapid and sensitive detection of genome contamination at scale with FCS-GX. *Genome Biol.* **25**, 60 (2024). 38409096 10.1186/s13059-024-03198-7 PMC10898089

[15] Mirchandani, C. *et al.**Plate Scale Tn5 Based Tagmentation Library Prep Protocol v1*. Preprint at 10.17504/protocols.io.4r3l2qmzpl1y/v1 (2024).

[16] Tarasov, A., Vilella, A. J., Cuppen, E., Nijman, I. J. & Prins, P. Sambamba: Fast processing of NGS alignment formats. *Bioinformatics* **31**, 2032–2034 (2015). 25697820 10.1093/bioinformatics/btv098 PMC4765878

[17] Li, H. Minimap2: Pairwise alignment for nucleotide sequences. *Bioinformatics* **34**, 3094–3100 (2018). 29750242 10.1093/bioinformatics/bty191 PMC6137996

[18] Chen, Z., Erickson, D. L. & Meng, J. Polishing the Oxford Nanopore long-read assemblies of bacterial pathogens with Illumina short reads to improve genomic analyses. *Genomics* **113**, 1366–1377 (2021). 33716184 10.1016/j.ygeno.2021.03.018

[19] Manni, M., Berkeley, M. R., Seppey, M., Simão, F. A. & Zdobnov, E. M. BUSCO update: Novel and streamlined workflows along with broader and deeper phylogenetic coverage for scoring of eukaryotic, prokaryotic, and viral genomes. *Mol. Biol. Evol.* **38**, 4647–4654 (2021). 34320186 10.1093/molbev/msab199 PMC8476166

[20] Seemann, T. Prokka: Rapid prokaryotic genome annotation. *Bioinforma. Oxf. Engl.* **30**, 2068–2069 (2014). 10.1093/bioinformatics/btu153 24642063

[21] Tatusova, T. *et al.* NCBI prokaryotic genome annotation pipeline. *Nucl. Acids Res.* **44**, 6614–6624 (2016). 27342282 10.1093/nar/gkw569 PMC5001611

[22] Grant, J. R. *et al.* Proksee: In-depth characterization and visualization of bacterial genomes. *Nucleic Acids Res.* **51**, W484–W492 (2023). 37140037 10.1093/nar/gkad326 PMC10320063

[23] Kaur, R. *et al.* Living in the endosymbiotic world of *Wolbachia*: A centennial review. *Cell Host Microbe* **29**, 879–893 (2021). 33945798 10.1016/j.chom.2021.03.006 PMC8192442

[24] Emms, D. M. & Kelly, S. OrthoFinder: Phylogenetic orthology inference for comparative genomics. *Genome Biol.* **20**(1). 10.1186/s13059-019-1832-y (2019). 10.1186/s13059-019-1832-y PMC6857279 31727128

[25] Daelemans, W., Van Den Bosch, A. & Weijters, T. IGTree: Using Trees for Compression and Classification in Lazy Learning Algorithms. In *Lazy Learning 407–423* (ed. Aha, D. W.) (Springer, Netherlands, 1997).

[26] Darling, A. C. E., Mau, B., Blattner, F. R. & Perna, N. T. Mauve: Multiple alignment of conserved genomic sequence with rearrangements. *Genome Res.* **14**, 1394–1403 (2004). 15231754 10.1101/gr.2289704 PMC442156

[27] Cabanettes, F. & Klopp, C. D-GENIES: Dot plot large genomes in an interactive, efficient and simple way. *PeerJ* **6**, e4958 (2018). 29888139 10.7717/peerj.4958 PMC5991294

[28] Almagro Armenteros, J. J. *et al.* SignalP 5.0 improves signal peptide predictions using deep neural networks. *Nat. Biotechnol.* **37**(4), 420–423 (2019). 30778233 10.1038/s41587-019-0036-z

[29] Krogh, A., Larsson, B., von Heijne, G. & Sonnhammer, E. L. Predicting transmembrane protein topology with a hidden Markov model: Application to complete genomes. *J. Mol. Biol.* **305**, 567–580 (2001). 11152613 10.1006/jmbi.2000.4315

[30] Magliery, T. J. & Regan, L. Sequence variation in ligand binding sites in proteins. *BMC Bioinformatics* **6**, 240 (2005). 16194281 10.1186/1471-2105-6-240 PMC1261162

[31] Prigozhin, D. M. & Krasileva, K. V. Analysis of intraspecies diversity reveals a subset of highly variable plant immune receptors and predicts their binding sites. *Plant Cell* **33**, 998–1015 (2021). 33561286 10.1093/plcell/koab013 PMC8226289

[32] Duarte, E. H., Carvalho, A., López-Madrigal, S., Costa, J. & Teixeira, L. Forward genetics in *Wolbachia*: Regulation of *Wolbachia* proliferation by the amplification and deletion of an addictive genomic island. *PLoS Genet.* **17**, e1009612 (2021). 34143770 10.1371/journal.pgen.1009612 PMC8244876

[33] Baião, G. C., Schneider, D. I., Miller, W. J. & Klasson, L. The effect of *Wolbachia* on gene expression in *Drosophila* paulistorum and its implications for symbiont-induced host speciation. *BMC Genomics* **20**, 465 (2019). 31174466 10.1186/s12864-019-5816-9 PMC6555960

[34] Ote, M., Ueyama, M. & Yamamoto, D. *Wolbachia* protein TomO targets *nanos* mRNA and restores germ stem cells in *Drosophila sex-lethal* mutants. *Curr. Biol.* **26**, 2223–2232 (2016). 27498563 10.1016/j.cub.2016.06.054

[35] Brockhurst, M. A. *et al.* Running with the red queen: The role of biotic conflicts in evolution. *Proc. R. Soc. B Biol. Sci.* **281**, 20141382 (2014). 10.1098/rspb.2014.1382 PMC4240979 25355473

[36] Choi, J. Y. & Aquadro, C. F. The coevolutionary period of *Wolbachia* pipientis Infecting *Drosophila* ananassae and its impact on the evolution of the host germline stem cell regulating genes. *Mol. Biol. Evol.* **31**, 2457–2471 (2014). 24974378 10.1093/molbev/msu204 PMC4137719

[37] Flores, H. A., Bubnell, J. E., Aquadro, C. F. & Barbash, D. A. The *Drosophila* bag of marbles gene interacts genetically with *Wolbachia* and shows female-specific effects of divergence. *PLOS Genet.* **11**, e1005453 (2015). 26291077 10.1371/journal.pgen.1005453 PMC4546362

[38] Martin, M., López-Madrigal, S. & Newton, I. L. G. The *Wolbachia* WalE1 effector alters *Drosophila* endocytosis. *PLoS Pathog.* **20**, e1011245 (2024). 38547310 10.1371/journal.ppat.1011245 PMC11003677

